# Ebola Virus Causes Intestinal Tract Architectural Disruption and Bacterial Invasion in Non-Human Primates

**DOI:** 10.3390/v10100513

**Published:** 2018-09-20

**Authors:** Ronald B. Reisler, Xiankun Zeng, Christopher W. Schellhase, Jeremy J. Bearss, Travis K. Warren, John C. Trefry, George W. Christopher, Mark G. Kortepeter, Sina Bavari, Anthony P. Cardile

**Affiliations:** 1 US Army Medical Research Institute for Infectious Diseases, 1425 Porter St., Fort Detrick, MD 21702, USA; ronald.b.reisler.ctr@mail.mil (R.B.R.); xiankun.zeng.fn@mail.mil (X.Z.); christopher.w.schellhase.mil@mail.mil (C.W.S.); jeremy.j.bearss.mil@mail.mil (J.J.B.); travis.k.warren.ctr@mail.mil (T.K.W.); sina.bavari.civ@mail.mil (S.B.); 2 Bacterial Respiratory and Medical Countermeasures Branch, US Food and Drug Administration, 10903 New Hampshire Avenue, Silver Spring, MD 20993, USA; john.trefry@fda.hhs.gov; 3 Joint Program Management Office, Medical Countermeasure Systems, 1564 Freedman Drive, Fort Detrick, MD 21702, USA; george.w.christopher.civ@mail.mil; 4 University of Nebraska Medical Center, College of Public Health, 42nd and Emile, Omaha, NE 68198, USA; mark.kortepeter@unmc.edu

**Keywords:** Ebola virus, intestinal tract, rhesus macaque, *Macaca mulatta*, kikwit, necrosis, hemorrhage, bacterial translocation, antibiotics

## Abstract

In the 2014–2016 West Africa Ebola Virus (EBOV) outbreak, there was a significant concern raised about the potential for secondary bacterial infection originating from the gastrointestinal tract, which led to the empiric treatment of many patients with antibiotics. This retrospective pathology case series summarizes the gastrointestinal pathology observed in control animals in the rhesus EBOV-Kikwit intramuscular 1000 plaque forming unit infection model. All 31 Non-human primates (NHPs) exhibited lymphoid depletion of gut-associated lymphoid tissue (GALT) but the severity and the specific location of the depletion varied. Mesenteric lymphoid depletion and necrosis were present in 87% (27/31) of NHPs. There was mucosal barrier disruption of the intestinal tract with mucosal necrosis and/or ulceration most notably in the duodenum (16%), cecum (16%), and colon (29%). In the intestinal tract, hemorrhage was noted most frequently in the duodenum (52%) and colon (45%). There were focal areas of bacterial submucosal invasion in the gastrointestinal (GI) tract in 9/31 (29%) of NHPs. Only 2/31 (6%) had evidence of pancreatic necrosis. One NHP (3%) experienced jejunal intussusception which may have been directly related to EBOV. Immunofluorescence assays demonstrated EBOV antigen in CD68+ macrophage/monocytes and endothelial cells in areas of GI vascular injury or necrosis.

## 1. Introduction

The clinical presentations reported during the 2014–2016 West Africa Ebola outbreak often included gastrointestinal (GI) symptoms: Abdominal pain, nausea, vomiting, and diarrhea [1,2,3,4]. Some clinicians reported copious amounts of vomiting and “cholera-like” levels of diarrhea during the outbreak [2,5,6]. This observation raises questions whether GI symptoms result from a primary gastroenteritis, or from a secondary complication due to systemic viral infection and whether current animal models accurately recapitulate human GI tract disease.

Many filovirus supportive care protocols include empiric antibiotics to cover the potential for gram-negative bacteremia of GI origin [7,8,9,10]. The use of empiric antibiotics seems to be supported by Kreuels et al. [5] who reported that their critically ill Ebola patient experienced intestinal dysmotility and sepsis presumably due to translocation of a multi-drug resistant (MDR) gram-negative bacillus from the GI tract. A recent study demonstrated bacterial sequences in 99/99 (100%) serum samples examined from Ebola patients [11]. Furthermore, Carroll et al. found that the majority of their cohort of 179 subjects that tested qRT-PCR positive for Ebola virus (EBOV) had evidence of bacterial translocation across the gut [12].

Very limited data exist regarding human pathology due to biosafety risks to personnel, the occurrence of disease in remote areas, the sporadic occurrence of outbreaks, and due to poor or limited surveillance [13]. In 2003, Geisbert et al. reported on the GI pathology observed in four cynomolgus macaques in a 1000 plaque forming unit (PFU) Ebola Zaire model [14]. Martines et al. [13] presented limited GI pathology data in their recent filovirus human and animal pathology review. Despite large numbers of infected individuals during the 2014–2016 outbreak, there have thus far been no autopsy studies performed or published. In light of these observations and the findings of Veazey et al. noting that the GI tract of rhesus macaques is remarkably similar to that of humans [15], we elected to conduct a systematic retrospective review of gastrointestinal pathology in the rhesus macaque intramuscular (IM) 1000 plaque forming unit (PFU) EBOV-Kikwit model.

## 2. Materials and Methods

Animal research at U.S. Army Medical Research Institute of Infectious Diseases (USAMRIID) is conducted under Institutional Animal Care and Use Committee (IACUC) approved protocols in compliance with the Animal Welfare Act, PHS Policy, and other federal statutes and regulations relating to animals and experiments involving animals. The facility where this research was conducted is accredited by the Association for Assessment and Accreditation of Laboratory Animal Care, International, and adheres to principles stated in the Guide for the Care and Use of Laboratory Animals, National Research Council, 2011.

### 2.1. Animal Use and Viral Challenge

The animals described in this manuscript served as control animals in therapeutic studies conducted at USAMRIID between 2014 and 2015 and had complete gastrointestinal tract pathology analysis. Existing data from these studies were analyzed retrospectively. These NHP studies were all performed by USAMRIID researchers utilizing the same institutional standard operating procedures and the same target viral challenge dose of 1000 PFU intramuscular (IM). Methods for NHP Ebola Virus (EBOV) challenge have been described in detail previously [16]. A total of 31 rhesus macaques, *Macaca mulatta* from seven separate studies, were inoculated intramuscularly (IM) with a target dose of 1000 PFU of EBOV (back titration titer range: 78–1358 PFU). The EBOV Kikwit strain utilized was EBOV H.sapiens-tc/COD/1995/Kikwit-9510621; this virus was primarily the 7U (7 uridy­lyls) variant at the mRNA editing site. The first passage of the virus, designated virus seed pool 807223, was conducted at the U.S. Centers for Disease Control and Prevention (CDC) using Vero E6 cells. A second passage of virus, designated WRC000121, was conducted at the University of Texas Medical Branch. WRC000121 was transferred to USAMRIID and propagated on BEI-Vero E6 cells to produce the USAMRIID master seed stock, R4415 [17]. The same virus stock was used for all seven studies. NHP cohort characteristics are described in Table 1. All NHPs in this cohort succumbed to EBOV infection. Clinical observation sheets were examined to assess the frequency of potential clinical gastrointestinal signs. A full necropsy was performed on all animals that were found dead or euthanized during the study period. Tissues collected for partial necropsy varied among the 7 protocols, but included at a minimum the following organs, which served as the focus for this disease model study: Liver, spleen, lymphoid tissues (numerous lymph nodes and mucosa-associated lymphoid tissues), lung, kidney, and gastrointestinal tract. Gross pathology lesion descriptions included in this study were derived from the individual animal necropsy checklists completed for each animal during necropsy.

All collected tissues were fixed in 10% neutral buffered formalin for at least 21 days. The tissues were trimmed and processed IAW USAMRIID Histology Laboratory SOPs. In brief, histology sections were cut at 5–6 µm on a rotary microtome, mounted on glass slides, and stained with hematoxylin and eosin IAW Histology Laboratory SOPs. Histologic lesions included in this study were derived from individual animal reports generated by a USAMRIID pathologist completed at the time of slide reading/report generation.

### 2.2. In Situ Hybridization

In situ hybridization (ISH) probe targeting the genomic EBOV NP gene was designed and synthesized by Advanced Cell Diagnostics (Cat# 448581, Newark, CA, USA). ISH was performed using the RNAscope 2.5 HD RED kit (Advanced Cell Diagnostics) according to the method described previously [18].

### 2.3. Immunofluorescence Assay

Detection of EBOV GP antigen in CD68+ cells and smooth muscle cells in the gastrointestinal tract of selected NHPs used an immunofluorescence assay (IFA) method. Formalin-fixed paraffin embedded (FFPE) tissue sections were deparaffinized using xylene and a series of ethanol washes. After 0.1% Sudan black B (Sigma, St. Louis, MO, USA) treatment to eliminate the autofluorescence background, the sections were heated in citrate buffer (pH 6.0) for 15 min to reverse formaldehyde crosslinks. After rinses with PBS (pH 7.4), the section was blocked with PBS containing 5% normal goat serum overnight at 4 °C. Then the sections were incubated with rabbit anti-EBOV GP polyclonal antibody (1:1500, USAMRIID) rabbit anti-CD31 polyclonal antibody (1:100, Abcam, ab28364, Cambridge, UK), mouse anti-CD68 monoclonal antibody (1:100, Dako Agilent Pathology Solutions, Clone KP1, Santa Clara, CA, USA), mouse anti-alpha smooth muscle actin (SMA) monoclonal antibody (1:400, R&D Systems, MAB1420, Minneapolis, MI, USA), and/or mouse anti-EBOV GP1,2 monoclonal antibody (1:400, USAMRIID, 6D8-1-241) for 2 h at room temperature [18]. After rinses with PBS, the sections were incubated with secondary Alexa Fluor 568 conjugated goat anti-rabbit and Alexa Fluor 488 conjugated goat anti-mouse antibody for 1 h at room temperature. Sections were cover slipped using the Vectashield mounting medium with DAPI (Vector Laboratories, Burlingame, CA, USA). Images were captured on a Zeiss LSM 880 confocal system (Zeiss, Oberkochen, Germany) and processed using open-source ImageJ software (National Institutes of Health).

## 3. Results

**Figure 1:**
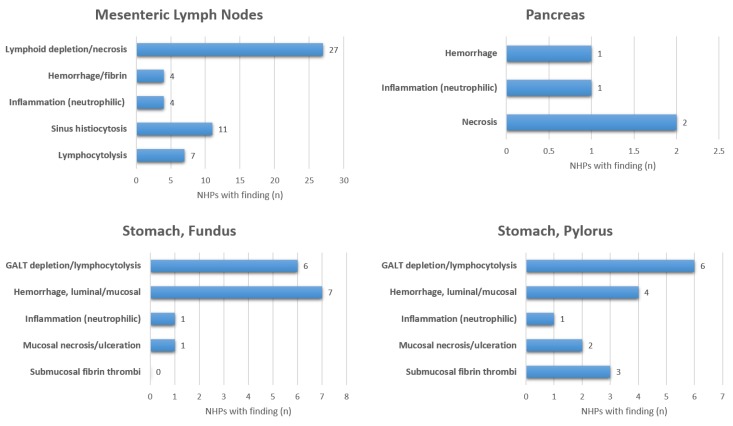
Summary of histopathologic findings for mesenteric lymph nodes, pancreas, and stomach in Ebola challenged rhesus macaques (*n* = 31).

**Figure 2:**
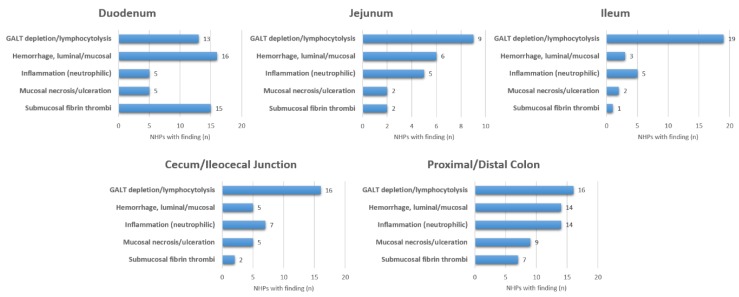
Summary of histopathologic findings for the duodenum, jejunum, ileum, cecum/ileocecal junction, and colon in Ebola challenged rhesus macaques (*n* = 31).

Eighteen of 31 (58%) of NHPs had clinical observation sheets available with descriptions of potential gastrointestinal clinical signs. Of those, two NHPs were observed to have emesis (11%), four had diarrhea (22%), and five had evidence of hematochezia (28%). The most commonly observed clinical sign was the development of anorexia (as defined by decreased biscuit consumption) in 16 NHPs (89%). Detailed GI pathology assessments are summarized in Figure 1 and Figure 2. All 31 NHPs exhibited lymphoid depletion of gut-associated lymphoid tissue (GALT) but the severity and the specific location of lymphoid depletion varied. The vast majority of NHPs experienced mesenteric lymphoid depletion and necrosis 27/31 (87%), but the extent of pathological findings varied by the segment of GI tract (Figure 1 and Figure 2). For example, GALT depletion and lymphocytolysis ranged from 6/31 (19%) observed in the stomach to 19/31 (61%) in the ileum. There was minimal pancreatic pathology observed in this cohort with only 2/31 (6%) demonstrating signs of pancreatic necrosis. One NHP with pancreatic necrosis and hemorrhage was euthanized at eight days post-infection (DPI) and the other NHP with pancreatic necrosis was euthanized at 15 DPI.

**Figure 3:**
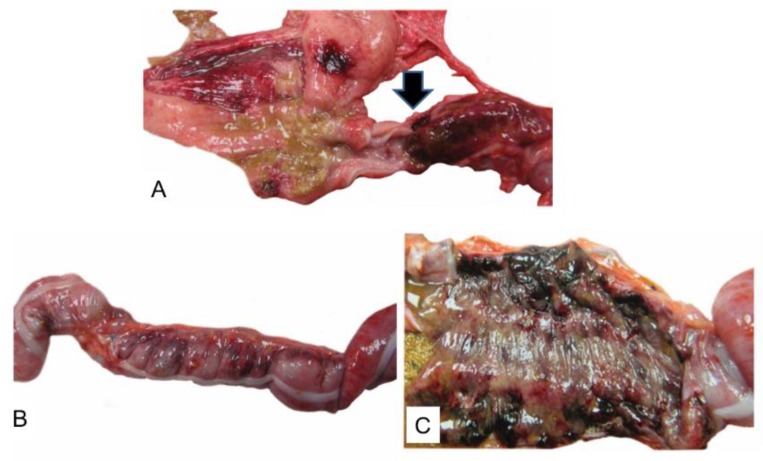
(**A**–**C**) Representative gross pathology images. (**A**) Stomach and duodenum, gross photo. There are multifocal hemorrhages of the gastric pyloric mucosa (left). Beginning distal to the pyloric sphincter, there is transmural hemorrhage and necrosis of the proximal duodenum likely due to infarction. (**B**) Colon, gross photo. Multifocal mural hemorrhages and congestion are visible from the external surface of the colon. (**C**) Colon, mucosa, gross photo. There are multifocal to coalescing areas of hemorrhage and necrosis visible in the colonic mucosa.

At gross necropsy and/or on histologic examination, partial to transmural necrosis and/or mucosal ulceration in the gastrointestinal tract (stomach, small intestine, and/or large intestine) was noted in 17/31 (55%) animals, and some NHPs demonstrated areas of necrosis and ulceration in multiple segments of the GI tract. Typical gross necropsy lesions observed in the gastrointestinal tract included mural and mucosal hemorrhage and congestion, often with luminal hemorrhage in the stomach and one or more segments of small and/or large intestine (Figure 3).

**Figure 4:**
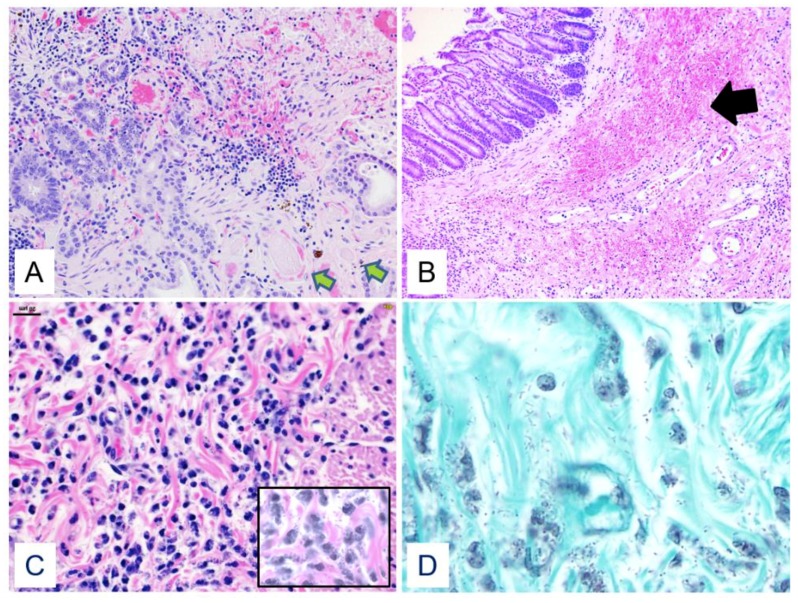
(**A**–**D**) **A**. Duodenum, macaque, HE, 200×. In an area of mucosal necrosis, there are numerous intravascular fibrin thrombi in submucosal blood vessels (green arrows). **B**. Distal colon, macaque, HE, 40×. There is focally extensive hemorrhage and edema in the submucosa with an inflammatory infiltrate (arrow). **C**. Colon, submucosa, macaque, HE, 400×. High power magnification of the submucosa from **C** shows bacilli visible on HE staining that are surrounded by neutrophils and occasionally within the cytoplasm (inset, 600×). **D**. Colon, submucosal connective tissue, macaque, Gram’s stain, 1000×. By Gram’s stain, bacilli in the submucosa are gram-negative.

**Figure 5:**
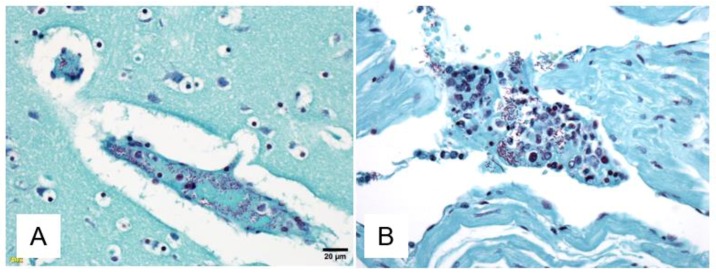
(**A**,**B**). Gram stain of tissues demonstrating evidence of gram-negative bacteremia in two different NHPs. (**A**) Demonstrates pathological evidence of disseminated gram-negative bacteria in the thalamus, 400×. (**B**) Demonstrates pathological evidence of disseminated gram-negative bacteria in the right heart, 400×.

Figure 4 shows histologic findings in the gastrointestinal tract of two different animals. 9/31 (29%) NHPs demonstrated evidence of focal areas of bacterial submucosal invasion in the GI tract. For these 9 NHPs, mean survival was 7.6 DPI [range 5–11] which was similar (*p* > 0.05) to the overall cohort’s mean survival time (8.3 DPI) and the mean log10 viral load at 5 DPI was identical to the overall cohort (*p* > 0.05). Of the nine NHPs with evidence of bacterial submucosal invasion, three NHPs had presumptive evidence of disseminated gram-negative bacterial infection, with gram-negative bacilli observed in tissue other than the GI tract (Figure 5).

**Figure 6:**
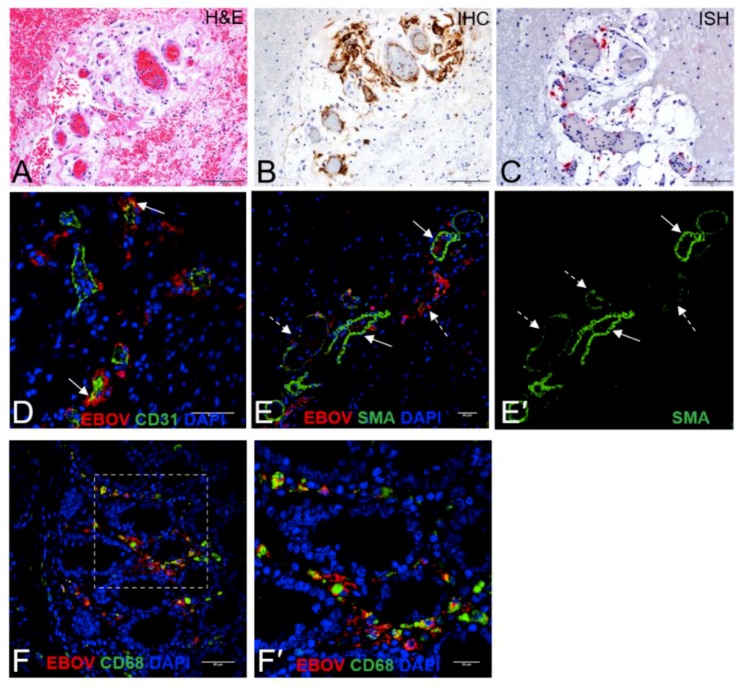
(**A**–**F**) Potential vascular origin Ebola virus (EBOV) infection in the colon. (**A**) In an area of submucosal hemorrhage, there is extravascular fibrin, perivascular edema and a mild infiltrate of neutrophils and mononuclear cells surrounding veins and small arteries. (**B**) By immunohistochemistry, vascular endothelial cells and some mononuclear cells are positive for EBOV glycoprotein (GP) antigen (brown), and there are scattered foci of intra- and extravascular antigen. (**C**) Intra- and extravascular EBOV genomic RNA (red) was detected in vascular endothelial cells and in monocytes and tissue macrophages. (**D**) EBOV GP (red) was detected in the areas surrounding the blood vessel (green) as well in vascular endothelial cells (arrow). (**E**) Extravascular EBOV GP (red) is present in the areas surrounding blood vessels (green). **E**,**E**’. Extravascular EBOV GP (red) is most evident in the perivascular spaces where smooth muscle actin (SMA, dashed arrow) staining is weak, suggestive of injury to the vascular wall, as opposed to areas with high levels of SMA expression (arrow) and little EBOV GP. (**F**,**F′**) By immunofluorescence there is abundant EBOV GP (red) associated with CD68+ macrophages/monocytes (green) in the colonic mucosa. **F′** is inset of **F** (white dashed rectangle) at high magnification. Scale bars, 100 μm in **A**–**C** and F, 50 μm in **D**–**F**, and 20 μm in **F′**.

Figure 4 demonstrates a typical lesion in the colon with hemorrhage, fibrin thrombi, ulcer, necrosis, and numerous bacilli, with viral antigen noted in the base of this GI ulcer. To further demonstrate how EBOV interacts histologically in the GI tract we included a hematoxylin and eosin (H and E), immunohistochemistry (IHC), ISH, smooth muscle actin (SMA)/EBOV glycoprotein (GP) immunofluorescence assay (IFA), CD31/EBOV glycoprotein (GP) IFA, and CD68/EBOV GP IFA staining of one of the three NHPs that demonstrated gram-negative bacterial infection at necropsy (Figure 6). These findings are consistent with and may suggest disruption of the epithelial barrier and degeneration of vascular endothelial cells, viral antigen in vascular endothelial cells, monocytes, macrophages, and in perivascular spaces, and bacterial translocation into the lamina propria.

One NHP in the cohort that died at 7 DPI experienced jejunal intussusception in which approximately 12 cm of proximal jejunum “telescoped” into the more distal jejunum. Gross necropsy and histologic observations confirm this as an ante-mortem lesion. Other gross necropsy findings in this animal included grossly enlarged (~1.5–2×) mesenteric lymph nodes, a known predisposing risk factor for intussusception [19].

## 4. Discussion

In the rhesus IM EBOV-Kikwit model, EBOV is believed to spread from the initial muscle infection site via antigen presenting cells (APCs)–monocytes, macrophages, and dendritic cells to regional lymph nodes, likely via lymphatics and the bloodstream and then rapidly disseminates throughout the entire host [20]. EBOV RNA is detectable as early as 3 DPI and peaks at 5–7 DPI [21]. In this model, we found that GALT is significantly affected in terminal disease, with pathological changes observed in animals that succumbed as early as 5 DPI. Once EBOV infected monocytes, macrophages, and dendritic cells arrive via the bloodstream to GALT, EBOV has been found to induce lymphocyte apoptosis, natural killer cell apoptosis, and destruction of the supporting cells [20]. Evidence suggests that EBOV also directly infects endothelial cells leading to increased vascular permeability [22]. Infected cells then produce proinflammatory cytokines, chemokines, and tissue factor (factor III), which, in turn, promote vasodilation, vascular permeability, and necrosis [23]. Presumably, because GALT represents approximately 70% of the entire host immune system [24], rapid onset GALT depletion and GI lymphocytolysis could cause localized immunosuppression. We hypothesize that rapid and extensive GALT depletion and GI lymphocytolysis may contribute to the high morbidity and mortality seen in a subset of the overall Ebola virus disease (EVD) population.

A further complication to the direct assault by EBOV on monocytes, macrophages, dendritic cells, and endothelial cells is the appearance of foci of gastrointestinal necrosis and hemorrhage early on in infection as was described by Geisbert et al. in EBOV-Kikwit in Cynomolgus macaques [14]. We observed that 55% of the NHPs in our cohort exhibited at least one area of necrosis and ulceration in the GI tract. The focal areas of GI necrosis and ulceration may have been exacerbated by systemic EBOV manifestations consistent with poor perfusion, tissue hypoxia, and ischemia possibly secondary to hypotension, tissue edema/inflammation, and microvascular clots [25]. GI mucosal integrity is an important line of defense against commensal GI flora. In a significant subset of the cohort 29%, GI mucosal necrosis and ulceration in this setting may have facilitated the introduction of potentially pathogenic gram-negative bacteria into the lamina propria, which in the setting of impaired host immunity due to EVD may have, in turn, provided an opportunity for further dissemination of both gram-negative bacteria and EBOV infected macrophages into the peritoneal space and the systemic circulation. In fact, 3/31 (10%) of the cohort experienced disseminated gram-negative infection.

Although emesis and diarrhea are infrequently reported in NHP studies of EVD, previous NHP reports demonstrated gastrointestinal pathology following oral, conjunctival, aerosol, and intramuscular inoculation [14,26,27]. Findings have included serosal bleeding, congestion at the gastroduodenal junction, focal erosions and necrosis, hemorrhage and thrombosis of submucosa and lamina propria, and moderate to severe necrosis of GALT [14,27], with no clear correlation with the primary route of infection. Our findings underscore the role of GALT as a target tissue, and in addition demonstrate bacterial translocation in a subset of NHPs into the lamina propria. This supports a model of viremic seeding of the GI tract, followed by vasculitis and disseminated intravascular coagulation as part of a systemic inflammatory response [28], leading to mucosal injury, complicated by bacterial translocation and sepsis, independent of the route of viral inoculation.

In human EVD, both Ansumana et al. [29] and Damkjaer et al. [30] adopted a “one size fits all” approach in their West African facilities, wherein all in-patients were provided the same supportive care treatment protocol regardless of the degree of symptomatology. Their supportive care regimens included broad-spectrum antibiotics for all patients. No randomized studies have been performed to date comparing the inclusion of antibiotics vs. withholding antibiotics in human EBOV disease. However, Ansumana et al. and Damkjaer et al. both included broad-spectrum antibiotics and reported case fatality rates (CFRs) of 31% (*N* = 581) and 42% (*N* = 33) in PCR confirmed Ebola adult and pediatric patients, respectively. Ansumana et al. utilized IV ceftriaxone and metronidazole and Damkjaer et al. also utilized IV ceftriaxone + metronidazole. Kreuels et al. [5] reported that a patient with severe EVD and gram-negative bacterial sepsis presumably due to bacterial gastrointestinal translocation was treated successfully in Germany with supportive intensive care unit (ICU) care which included IV Fluids, IV antibiotics, and mechanical ventilation. This patient was started on broad-spectrum IV antibiotics on day two of illness after developing a significant fever, but received no antiviral therapies. In a review of the patients cared for in European and US facilities, 81% (22/27) of patients received empiric antibiotics, along with other intensive care measures and, in many cases, investigational products, which yielded an overall mortality of 18.5%, which was lower than that seen in West Africa; however, what specific measures led to the lower mortality cannot be determined [10].

The finding that pathological evidence of pancreatitis was relatively rare (6%) in the rhesus IM EBOV-Kikwit model was not surprising, given our previous observations that significant biochemical evidence of pancreatitis, elevations of serum lipase, were not observed in three NHP IM models: EBOV-Kikwit or EBOV-Makona in rhesus macaques and EBOV-Kikwit in Cynomolgus macaques [21]. While some authors have reported hyperamylasemia in human EBOV in the past [29], it is entirely possible that the hyperamylasemia observed in human EBOV cases may not have been a marker for pancreatic injury but may have been either salivary in origin, a marker of intestinal injury, or renal failure [31].

While animal models of disease have contributed a great deal to our understanding of human disease, there may be inherent differences in disease manifestation when comparing EVD in NHPs to humans [32,33]. No animal model perfectly recapitulates human disease. However, it is noteworthy that the EBOV 1000 PFU IM model in rhesus NHPs is under consideration by the US FDA as a reasonable animal model for drug development for EVD in humans under the animal rule. There are differences in the manner in which EVD is manifested clinically in NHPs and humans. One relevant example would be that in advanced EVD, few NHPs experience vomiting, and diarrhea, as illustrated by the data herein, whereas the majority of humans experience vomiting and diarrhea.

A key caveat to our analysis is that our data reflect a retrospective analysis of NHPs used as controls in seven different studies of IM administered EBOV. In addition, we are unable to comment regarding the species of gram-negative rod observed in the three animals with disseminated bacteria, other than it appears to have come from the GI tract—in that the morphology of the bacteria appears to be the same as the morphology of the disseminated bacteria. There were no blood cultures, tissue cultures, or PCR-based quantitative bacterial studies performed in these protocols. Of note, gram-negative bacteremia has been observed in humans infected with EBOV and it has been hypothesized that this is related to gut translocation [5,11,12]. Another limitation of our analysis is that 2/31 (6%) of the NHPs in our cohort were found dead. We acknowledge that the time from death to necropsy was above the mean for the two animals that were found dead but we do not believe that the pathological findings were significantly affected by the fact that they were found dead. While it is true that delays in necropsy can lead to increased numbers of pathological artifacts, they are distinguishable artifacts and our pathologists do not believe that our findings are significantly affected by the time from death to necropsy. Another limitation is that our analysis focused on the 1000 PFU IM EBOV Kikwit rhesus model. We are unable to comment specifically on potential findings and comparisons to other NHP EBOV models and EBOV viral strains (e.g., Mayinga and/or Makona). A final limitation is that we have not adopted a consensus GI pathology severity scoring system. Our analysis is retrospective and meant to be hypothesis generating. Adoption of a consensus GI pathology scoring system to be tested prospectively would be of benefit.

One of the NHPs in our series was found to have intussusception at necropsy. We are aware of only one previous report of intussusception in NHP models of EVD [34]. Although intussusception is recognized as a complication of infection due to other viruses (e.g., norovirus, rotavirus, adenovirus, astrovirus, herpes viruses, cytomegalovirus), its mechanism is not well understood but may be related to a peristaltic lead point created by mesenteric lymphadenopathy or a viral-mediated inflammatory neuropathy [35,36,37]. This NHP was somewhat unique compared to the other NHPs in our cohort as it experienced significant mesenteric lymphadenopathy in contradistinction to the typical lymphocytolysis that we observed. The pathological findings were non-specific in nature and did not provide us with any insight as to why this particular NHP experienced intussusception. In humans, intussusception left untreated has an exceedingly high mortality rate [19]. While there have been no reports to date of intussusception in human EVD, the vast majority of human cases are not imaged radiologically, nor are detailed autopsies performed; thus, we simply do not know the incidence in the human population. We believe that intussusception could very well be occurring in an unknown small percentage of human EVD cases and would anticipate that untreated intussusception coupled with EVD would result in a fatal outcome.

## 5. Conclusions

In summary, EBOV-infected humans often complain of severe abdominal pain, vomiting, and diarrhea at the time of clinical presentation [38,39]. EBOV related GI pathological changes in the NHP IM rhesus Kikwit model are extensive, occur as early as 5 DPI, and could impact the clinical disease course. Our findings support a model of viremic seeding of the GI tract, GI tract injury, and systemic inflammatory response, exacerbated by bacterial translocation and sepsis, and suggest that intussusception may be an under-recognized complication. These findings coupled with reports suggesting improved CFRs with supportive care during the 2014–2016 West Africa Ebola outbreak [8,29,30], that included empiric antibiotic therapy directed against gram-negative enteric microflora warrant further study in both NHP models and in future outbreaks of human disease.

## Figures and Tables

**Table 1:** Cohort characteristics.

Variable	(*N* = 31)
Mean weight, kg [range]	4.9 [3.8–7.3]
Mean age, months [range]	48.3 [35–79]
Male:Female ratio	15:16
Day 5 Post-infection (log 10 copies/mL)	8.68
Survival time (days) [range]	8.0 [5–15]
Found Dead	6%
Mean Time from Death to Necropsy (minutes) [range]	409.8 [25–2278]
NHPs showing evidence of bacterial invasion of intestinal tract tissue (gram-negative rods)	29%
